# Differences on Brain Connectivity in Adulthood Are Present in Subjects with Iron Deficiency Anemia in Infancy

**DOI:** 10.3389/fnagi.2017.00054

**Published:** 2017-03-07

**Authors:** Cecilia Algarin, Keerthana Deepti Karunakaran, Sussanne Reyes, Cristian Morales, Betsy Lozoff, Patricio Peirano, Bharat Biswal

**Affiliations:** ^1^Sleep and Neurofunctional Laboratory, Institute of Nutrition and Food Technology, University of ChileSantiago, Chile; ^2^Department of Biomedical Engineering, New Jersey Institute of Technology, University HeightsNewark, NJ, USA; ^3^Center for Human Growth and Development, University of MichiganAnn Arbor, MI, USA

**Keywords:** iron deficiency anemia, infancy, long-lasting effects, brain connectivity, resting state networks, default mode network

## Abstract

Iron deficiency continues to be the most prevalent micronutrient deficit worldwide. Since iron is involved in several processes including myelination, dopamine neurotransmission and neuronal metabolism, the presence of iron deficiency anemia (IDA) in infancy relates to long-lasting neurofunctional effects. There is scarce data regarding whether these effects would extend to former iron deficient anemic human adults. Resting state functional magnetic resonance imaging (fMRI) is a novel technique to explore patterns of functional connectivity. Default Mode Network (DMN), one of the resting state networks, is deeply involved in memory, social cognition and self-referential processes. The four core regions consistently identified in the DMN are the medial prefrontal cortex, posterior cingulate/retrosplenial cortex and left and right inferior parietal cortex. Therefore to investigate the DMN in former iron deficient anemic adults is a particularly useful approach to elucidate de long term effects on functional brain. We conducted this research to explore the connection between IDA in infancy and altered patterns of resting state brain functional networks in young adults. Resting-state fMRI studies were performed to 31 participants that belong to a follow-up study since infancy. Of them, 14 participants were former iron deficient anemic in infancy and 17 were controls, with mean age of 21.5 years (±1.5) and 54.8% were males. Resting-state fMRI protocol was used and the data was analyzed using the seed based connectivity statistical analysis to assess the DMN. We found that compared to controls, former iron deficient anemic subjects showed posterior DMN decreased connectivity to the left posterior cingulate cortex (PCC), whereas they exhibited increased anterior DMN connectivity to the right PCC. Differences between groups were also apparent in the left medial frontal gyrus, with former iron deficient anemic participants having increased connectivity with areas included in DMN and dorsal attention networks. These preliminary results suggest different patterns of functional connectivity between former iron deficient anemic and control young adults. Indeed, IDA in infancy, a common nutritional problem among human infants, may turn out to be important for understanding the mechanisms of cognitive alterations, common in adulthood.

## Introduction

Iron deficiency anemia (IDA) continues to be the world most prevalent micronutrient deficit. Its frequency is greater in children and pregnant women and can affect up to 70% of population in many countries, particularly economically developing countries (Swaminathan et al., 2013). Even in developed countries, the prevalence of IDA in children can be as high as 4% (Jáuregui-Lobera, 2014). In Chile, until as late as 1999, about 30% of the children under 2 years of age presented with IDA, often as a result of poor maternal health condition or poor dietary intake. Public health policies of “*fortification”* such as availability of formula milk at government health centers, caused prevalence of IDA in infants under 18 months of age to drop to 10% in a period of 10 years (Brito et al., 2013). Although the decreasing incidence is promising, it should be emphasized that iron is an important nutrient for brain development, and the impact of IDA in infancy development, even after iron therapy is of clinical relevance. Therefore, understanding the neural mechanisms through which the developing brain is coping with a nutrient deficiency may help improve therapeutic strategies aimed at optimizing brain functional integrity.

Studies using rodent models indicate that early IDA modifies myelin protein profile, specifically the proteolipid protein and MBP 21 (myelin basic protein), both of them required for myelin compaction (Ortiz et al., 2004). Further, alteration in genes regulating dendritic morphology appears to alter experience-depending brain synaptic plasticity, derailing thus memory, learning and other developmental milestones (Georgieff, 2008).

In line with these results, there is mounting evidence that IDA in human infants is associated with long-lasting negative outcomes on several neurofunctional and cognitive domains, extending into adolescence (Algarín et al., 2013). We have shown that children who were properly treated for IDA during infancy, had delayed latencies in auditory and visual evoked potentials at preschool age. This slower neuronal transmission in both sensory systems is consistent with an impaired myelination process (Algarín et al., 2003).

Animal models have shown that early iron deficiency modifies the dopaminergic neurotransmission system, altering the functioning of the hippocampus and frontal cortex, among other brain regions (Beard and Connor, 2003). Moreover in rats, dopamine synaptic connections develop quickly during the first 15 days of post-natal life; iron deficiency throughout this period would likely influence these synaptic connections (Ward et al., 2007). Dopaminergic neurotransmission system is critically involved in executive functions which encompass a heterogeneous repertoire of abilities related to monitoring and controlling thought and action, including self-regulation, inhibitory motor, cognitive control, planning future actions, attentional flexibility and error detection and correction capacity, among others (Prencipe et al., 2011; Lantrip et al., 2016). These higher cognitive functions have been well studied in humans using both event-related potentials (ERPs) and functional magnetic resonance imaging (fMRI).

Using ERPs, measure brain electrical activity when a subject performs a task, we found that at 10 years of age, former IDA (FIDA) children exhibited longer reaction time and lower P300 amplitude in the Go/No-Go task, which evaluates inhibitory motor control (Algarín et al., 2013). These results are consistent with subsequent research using a recognition memory paradigm (also at 10 years of age) that found slower reaction time and smaller ERP response to new/old tasks with an almost equal level of performance accuracy, suggesting that the effects of IDA in infancy persisted into late childhood despite apparently normal behavior (Congdon et al., 2012).

Neurophysiological and imaging assessments are complementary methods for the study of brain functions. There has been much progress regarding typical and atypical human brain activities at the group level using fMRI and resting state fMRI (rs-fMRI; Dosenbach et al., 2010). Resting state networks (RSN) are defined by a set of functionally interacting spatially distant regions at rest (Biswal, 2012). The networks are known to be organized in different functional systems that correlate to behavioral performance, allowing for further understanding of the significance of functional connectivity. Several studies on the developing brain show that the extent of brain regions exhibiting resting state functional connectivity increases from 2 weeks of age up to 2 years old (Lin et al., 2008). Fair et al. (2008) showed that functional connectivity between ventro-medial prefrontal cortex and posterior cingulate cortex (PCC) differs among children, adolescents and young adults, whereas, regions involved with motor and conflict monitoring differs little throughout these periods. These results indicate that the motor system and goal directed behavior develop earlier than other cognitive systems. A recent report from Marek et al. (2015) showed that network connectivity decreases in early adolescence (13–15 years) and then increases from late adolescence to adulthood, with the exception of the link between two of the most recognized networks, the Default Mode Network (DMN) and fronto-parietal network.

An important characteristic regarding the RSN, is the strength of the correlation between functional and structural connectivity. From rodent model, the results of the study conducted by Hübner et al. (2017) inducing brain demyelination, showed that functional connectivity was also altered, and the areas most affected were the posterior centers of DMN. In humans, myelination and functional connectivity develop parallel from birth until late adolescence (Stevens et al., 2009). Greicius et al. (2009) showed that there was not functional connectivity between hemispheres in absence of corpus callosum, reinforcing the concept that structural neural connections are required for functional connectivity. This hypothesis is further supported by studies showing that the strength of functional and structural connectivity is correlated in both healthy and patients with cognitive dysfunction (Hagmann et al., 2010). As an example, clinical evidence of the interaction between myelin disorder and functional connectivity alteration has been reported in patients with Multiple Sclerosis; subjects with an active lesion have increased functional connectivity that might be secondary to a compensatory mechanism for limiting and repairing the injury (Droby et al., 2016).

Although there is some data regarding long-lasting cognitive effects in FIDA human adults (Lukowski et al., 2010), to our knowledge, there are no studies using rs-fMRI studies. Given that the DMN is a core brain network, the assessment of DMN in FIDA adults is particularly imperative in order to understand the long-lasting effects of IDA in infancy on neural functioning in adulthood. To achieve this, we used resting state fMRI to compare baseline whole-brain connectivity in both groups. We hypothesized that FIDA participants will present altered brain networks connectivity relative to control participants.

## Materials and Methods

Participants were part of a longitudinal cohort study, which began recruitment in 1990 in order to conduct research on the behavioral, neuro-functional development and sleep-wake patterns effects of IDA in infancy. Six cognitive follow-ups were conducted at 12 and 18 months, 3, 5, 10 and 16 years old. The follow-ups included, among other measurements, cognitive assessment, academic achievements, socioeconomical and nutritional status. In brief, inclusion criteria were healthy full-term infants with birth weights ≥3.0 kg, no perinatal complications, and no acute or chronic illnesses. Infants were assessed for IDA at 6, 12 and 18 months. Anemia was defined as venous hemoglobin ≤100 g/L at 6 months and <110 g/L at 12 and 18 months. Iron deficiency was defined as at least two out of three iron measures in the iron-deficient range (mean cell volume <70 fl, erythrocyte protoporphyrin >100 μg/dL red blood cells [1.77 μmol/L], serum ferritin <12 μg/L). For each IDA infant, a non-anemic (venous hemoglobin ≥115 g/L) infant of the same age was randomly selected, constituting the “control group”. After initial testing, all infants were given supplemental iron for 6–12 months (Lozoff et al., 2003). No participant presented IDA thereafter.

In year 2014, all the cohort members who participated in the previous follow-ups and were approximately 22 years old were invited to perform a brain magnetic resonance. The present study involved 31 healthy young adult participants (17 controls and 14 FIDA) with a mean age of 21.5 years (±1.5 years), and 55% were male.

For current and background characteristics of the subjects we compared the Graffar scale (socio-ecomomical status; Richaud et al., 2013), performed the Spanish version of Manual for the State-Trait Anxiety Inventory (STAI “Self-Evaluation Questionnaire”; Guillén-Riquelme and Buela-Casal, 2011), and established current occupation (employee, studying or unemployed).

The research protocol was approved by the Institutional Review Boards of the University of Michigan Medical Center, Ann Arbor, Institute of Nutrition and Food Technology (INTA), University of Chile, Santiago, and the Office of Protection from Research Risks, NIH. Participants signed an informed consent.

### Imaging Parameters

fMRI was performed using Siemens Magnetom Skyra 3T scanner with parameters at TR = 2000 ms, TE = 35 ms with 16, 7.040-mm slices, 1-mm slice gap, and an in-plane resolution of 2.553 × 2.553 mm^2^. Total of 200 time points were collected for every subject. For the anatomical T1 scan, imaging was performed on the same scanner with TR = 20 ms, TE = 4.92 ms with 303 slices and field of view (FOV) of 192 × 499 mm^2^.

### Preprocessing

For all participants functional and anatomical image preprocessing was performed using statistical parametric mapping software (SPM 8 implemented using Matlab R2012b). After manually performing anterior commissure alignment for each participant, a realignment step was accomplished to correct for head motion. During this step, all scans of the participant were realigned to their first scan of the first session. The details of the transformation in six directions (*x*, *y*, *z*, roll, pitch and yaw) were stored as translation and rotation parameters. All participants were within the acceptable head motion range (less than 2 mm). After realignment, functional images were co-registered to the anatomical image (T1-weighted image) of that participant. Following successful co-registration of anatomical and functional images, segmentation was performed with SPM8 toolbox, and a probability map of gray matter, white matter (WM) and cerebrospinal fluid (CSF) was obtained. Simultaneously, a field vector storing information on the transformation from native subject space to standard template (Montreal Neurologic Institute, MNI template) was calculated. For each participant, the functional image was transformed to MNI space based on the deformation field and re-sampled to 3 mm isotropic voxel size. A binary mask of WM and CSF was created when a threshold for the WM/CSF maps obtained from segmentation step at *p* > 0.98 was applied (Biswal et al., 2010). In order to remove the confounding effects of WM, CSF and head motion, they were included as nuisance variables in linear regression model. The nuisance variable for linear regression included five principal components of WM and CSF obtained from WM and CSF masks. The regression input also included six time series describing head motion in six directions, six time series describing head motion in previous time points and 12 time series describing quadratics of motion. The residual of linear regression was temporally bandpass filtered at 0.01–0.1 Hz and spatially smoothened using a Gaussian kernel of 6 mm full width at half maximum. This filtered and smoothened data was used for further analysis. Acquisition, processing and analysis were performed to whole brain.

### Seed Based Connectivity

To analyze the intrinsic functional connectivity of PCC with the whole brain, two spheres of 5 mm radius was generated and used as a seed for left and right PCC seeds using MNI coordinates. RSN were obtained from coherent fluctuations of 0.01–0.1 Hz frequency occurring at spatially distinct regions through underlying monosynaptic or polysynaptic anatomical connections. Therefore, such RSN can be derived using any seed within a network. Seeds of 5 mm radius were also generated for left and right medial frontal gyrus (MFG), another major component of DMN. For all four seeds, the average blood oxygen level-dependent (BOLD) time series was calculated for each seed region and a whole brain voxel wise Pearson’s r correlation was performed for each subject. Subject level correlation maps were later converted to z-maps using r-z Fisher transformation.

### Regions of Interest (ROI)-Based Connectivity

In addition to seed based correlation of the PCC and MFG, a total of 160 regions of interests (ROIs) were created based on the list defined by Dosenbach et al. (2010). All ROIs were sorted based on RSN’s: 1. Cerebellar, 2. Cingulo-Opercular, 3. Default mode, 4. Fronto-Parietal, 5. Occipital, and 6. Sensoriomotor networks. Using the predefined MNI coordinates, a 5 mm radius sphere was created surrounding the peak voxel sampled at the same voxel dimensions of participants BOLD fMRI image. Nine ROIs were discarded as they were not within the brain mask of at least one subject. Using the remaining 151 ROI as mask, an average BOLD signal for each ROI was calculated for all participants. Pearson’s r correlations were calculated between the BOLD signals of each ROI with remaining ROI. Hence a 151*151 correlation matrix was generated for each participant. Each 151*151 correlation matrix was transformed into Z-matrices using Fisher’s r-z transformation.

### Statistical Analysis

#### Seed Based analysis

The z-maps were concatenated to create two 4D functional z-maps, one for each group. An unpaired two-sample two tailed *t*-test was performed on the 4D inputs with gender as covariate using Functional MRI of the Brain Software Library randomized implementation. All statistical comparisons were performed only for voxels that were present across all subjects. Further, Monte-Carlo simulation was implemented to perform cluster based thresholding on the uncorrected voxel-wise p-maps to correct for multiple comparison problem.

#### ROI Based Connectivity

An independent sample *t*-test was performed on the ROI pairs to compare the connectivity between FIDA and control groups at *p* < 0.001. Due to the small sample size, the statistical threshold was set to lenient with no additional correction for multiple comparison problems.

## Results

Control and FIDA groups were similar in current characteristics, including socioeconomic status, emotion scales and gender (Table 1).

**Table 1:** **Characteristics of Control and FIDA groups**.

	Control (*n* = 17)	FIDA (*n* = 14)	*p*-value
Age (year)	21.3 (20.9–21.5)	21.2 (21.0–21.6)	0.150
Males (%)^a^	8 (47)	9 (64)	0.337
**Occupation**^b^			
Employed (%)	13 (42)	6 (19)	0.145
Student (%)	4 (13)	5 (16)	
Unemployed (%)	0 (0)	3 (10)	
**STAI scale**			
Negative emotion	29.1 (26.5–31.7)	26.0 (23.1–28.8)	0.925
Positive emotion	8.8 (7.3–10.2)	10.5 (8.8–12.1)	0.173
Anxiety	6.2 (5.0–7.4)	6.7 (5.4–8.0)	0.601
**Socioeconomic status**			
Graffar score (infancy)	28.6 (24.3–32.9)	28.9 (25.0–32.8)	0.916
Graffar score (5 years)	26.3 (22.9 29.7)	24.5 (21.4–27.6)	0.426
Graffar score (15 years)	34.0 (30.8–37.1)	33.6 (30.8–36.4)	0.865

*Abbreviations: FIDA, former iron-deficiency anemia; STAI, state-trait anxiety inventory. Values are expressed as mean (confidence intervals). T-test, ^a^chi square test, ^b^and estimating missing cell frequencies (Graf et al., 1997)*.

### Seed Based Connectivity Analysis

#### Posterior Cingulate Cortex

**Figure 1:**
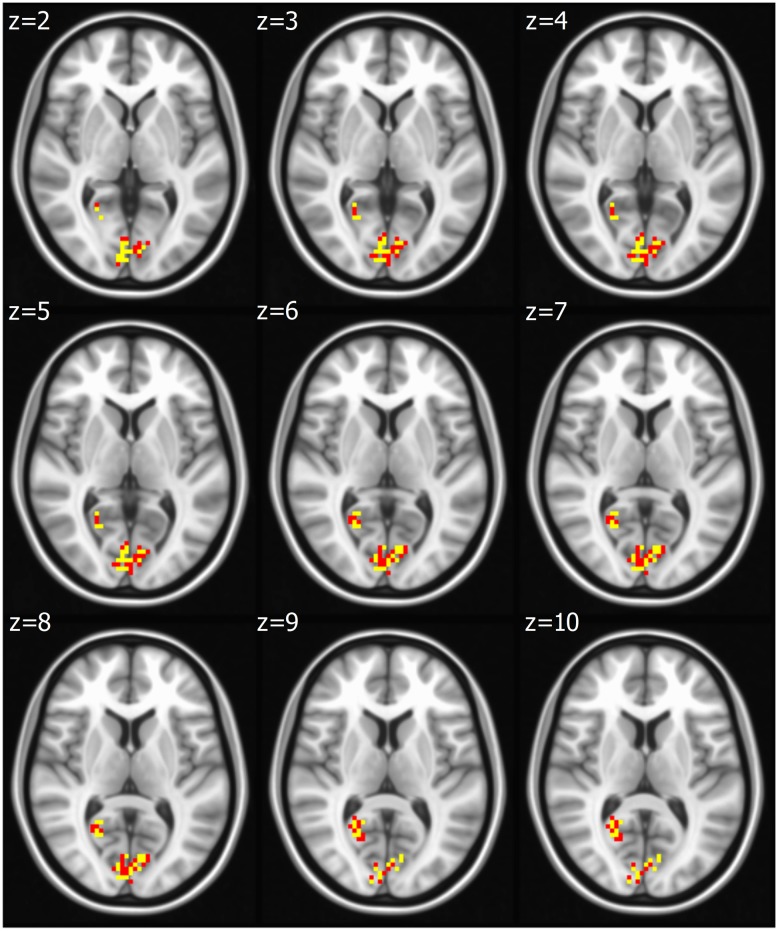
**Right posterior cingulate cortex (PCC) connectivity in Controls**. Right PCC connectivity greater in Control group. Red indicates regions with significance *p* < 0.02 and yellow indicates regions with significance *p* < 0.005.

**Figure 2:**
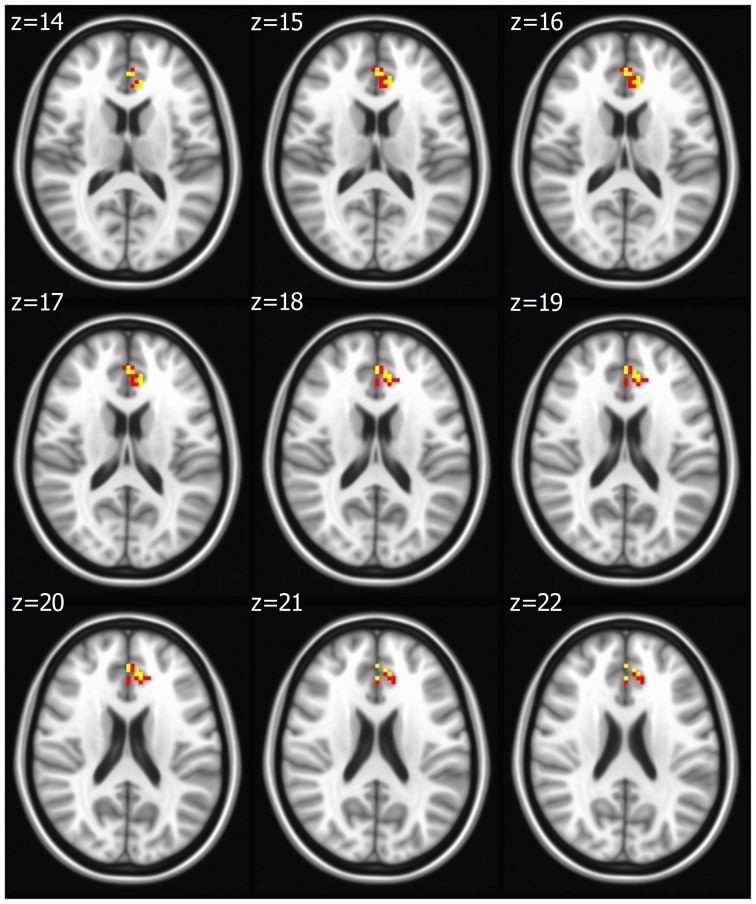
**Right PCC connectivity in former iron-deficiency anemia (FIDA)**. Right PCC connectivity greater in FIDA group. Red indicates regions with significance *p* < 0.02 and yellow indicates regions with significance *p* < 0.005.

**Figure 3:**
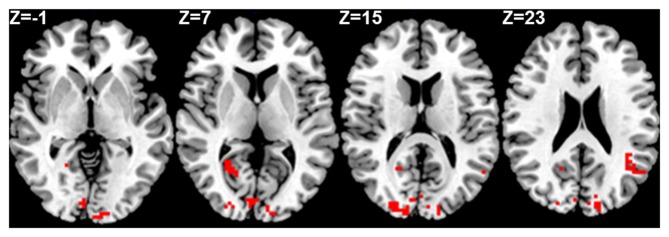
**Left PCC connectivity in Control group**. Red indicates regions with significance *p* < 0.005.

Seed based analysis of left and right PCC resulted in a connectivity map involving core anatomical regions of DMN, including bilateral ventral medial prefrontal cortex, posterior cingulate/retrosplenial cortex, lateral temporal cortex, dorsal medial prefrontal cortex and hippocampal formation. Randomization method performed on both left and right PCC z-maps resulted in voxel-based p-maps for contrast 1) Control > FIDA and 2) FIDA > Control. P-maps of right PCC thresholded at *p* < 0.02 with Monte Carlo cluster size = 38.9 voxels resulted in two clusters greater in Control (Figure 1 and Table 2) and one cluster greater in FIDA (Figure 2 and Table 2) participants. Similarly, randomization results of left PCC threshold at *p* < 0.02 with Monte Carlo cluster size = 40.3 voxels resulted in five clusters greater in Controls and no clusters greater in FIDA (Figure 3 and Table 3). Regions showing decreased connectivity to left and right PCC in FIDA group included regions of posterior DMN such as cuneus, PCC, parahippocampal gyrus, medial temporal gyrus and lingual gyrus. In contrast, regions presenting increased connectivity to right PCC in FIDA group included regions of anterior DMN and anterior cingulate cortex.

**Table 2:** **Connectivity to right posterior cingulate cortex (PCC) in Control and FIDA groups**.

Control group > FIDA group		
Cluster size (voxels)	Region	*X, Y, Z* coordinates in TLRC space
140 (36)	Right lingual gyrus (R.cuneus)	+3.0, −85.0, +1.0 (+6, −88, +11)
48	Right posterior cingulate cortex	+18.0, −58.0, +16.0
**FIDA group > Control group**		
**Cluster size (voxels)**	**Region**	***X, Y, Z* coordinates in TLRC space**
89 (19)	Left anterior cingulate cortex (L.ACC)	−3.0, +29.0, +7.0 (−9, +38, +16)

*List of clusters with significantly greater connectivity to right PCC (−10, +54, +14) in 1) Control group and 2) FIDA group at voxel wise threshold p < 0.02 with multiple comparison correction using cluster threshold = 38.9 voxels. Values within parenthesis indicate regions with significance (p < 0.005 at cluster size = 16.7)*.

**Table 3:** **Connectivity to left PCC in Control group**.

Control group > FIDA group		
Cluster size (voxels)	Region	*X, Y, Z* coordinates in TLRC space
61	Left cuneus	−15.0, −94.0, +6.0
46	Left cuneus	−0.0, −79.0, +19.0
46	Left middle temporal gyrus	−50.0, −58.0, +23.0
45	Right parahippocampal gyrus	+24.0, −52.0, +7.0
42	Right cuneus	+21.0, −91.0, +13.0

*List of clusters with significantly greater connectivity to left PCC (+10, +54, +14) in control group at voxel wise threshold *p* < 0.02 with multiple comparison correction using cluster threshold = 40.3 voxels*.

#### Medial Frontal Gyrus (MFG)

**Figure 4:**
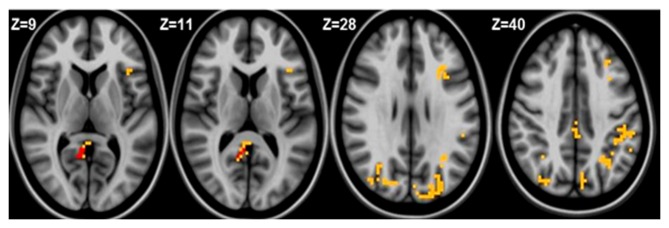
**Left medial frontal gyrus (MFG) connectivity in FIDA group**. Red indicates regions with significance *p* < 0.005.

Seed based analysis of left and right MFG resulted in a connectivity map involving core anatomical regions of DMN. Randomization method performed on both left and right MFG z-maps resulted in voxel-based p-maps for contrasts 1) Control > FIDA and 2) FIDA> Control. P-maps of both left and right MFG in the contrast Control > FIDA were not statistically significant after Monte Carlo cluster thresholding at *p* < 0.02. However, the FIDA > Control contrast condition of left MFG threshold at *p* < 0.02 with Monte Carlo cluster size = 38.3 voxels resulted in nine clusters greater in FIDA. The regions exhibiting increased connectivity to left MFG in FIDA group included DMN and dorsal attention networks. The list of regions and their coordinates mainly includes the intraparietal lobule and precuneus (Table 4 and Figure 4).

**Table 4:** **Connectivity to left medial frontal gyrus (MFG) in FIDA group**.

Cluster size (voxels)	Region	*X, Y, Z* coordinates in TLRC space
93	Left inferior parietal lobule	−50.0, −35.0, +28.0
77	Right cuneus	+27.0, −79.0, +21.0
73	Left middle frontal gyrus	−33.0, +23.0, +22.0
73	Left cuneus	−21.0, −91.0, +22.0
52 (20)	Right posterior cingulate cortex (R.PCC)	+12.0, −55.0, +16.0 (+12, −55, +16)
51	Left precuneus	−30.0, −61.0, +34.0
50	Left cuneus	−3.0, +82.0, +34.0
45	Left paracentral lobule	−3.0, −34.0, +49.0
41	Right superior parietal lobule	+33.0, −52.0, +58.0

*List of clusters with significantly greater connectivity to left medial frontal gyrus (+6, −47, −5) in FIDA group at voxel wise threshold *p* < 0.02 with multiple comparisons correction using cluster threshold = 39.4 voxels. Values within parenthesis indicate regions significant at *p* < 0.005 at cluster size = 16.7*.

### ROI Based Connectivity Analysis

**Figure 5:**
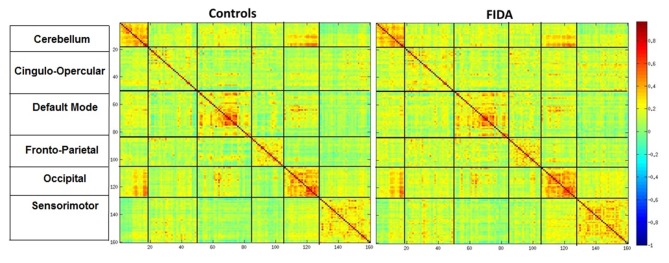
**Mean connectivity matrix**. Mean Connectivity matrix in Controls and FIDA groups. Color bar indicates Pearsons r correlation ranging from 1 to −1, hot colors indicating positive correlation between two regions. Vertical and horizontal partitions represent functional connectivity networks.

Control and FIDA groups showed greater connectivity within Cerebellum, DMN and Occipital networks as compared to between network connectivity. Based on the connectivity matrix, FIDA participants presented a scattered decrease in negative connectivity between Cingulo-opercular, Fronto-parietal and Sensory motor networks with DMN, and decrease in positive connectivity within DMN network. Otherwise, they exhibited a sparse increase in positive connectivity within Cingulo-opercular and Fronto-parietal networks (Figure 5).

## Discussion

These preliminary findings based on specific rs-fMRI assessments suggest different patterns of functional connectivity between FIDA and control young adults.

Seed based connectivity results show that FIDA participants have decreased connectivity of the left PCC with posterior DMN regions, relative to control participants. The PCC is a highly connected and metabolically active brain region and a component of several RSN. The link between PCC and DMN is of particular importance due to the possibility of the PCC’s role as a “hub” interacting with multiple brain networks to allocate cognitive resources to different tasks (Leech and Sharp, 2014).

Our findings are reminiscent of DMN patterns observed in patients with neurodegenerative diseases, as Alzheimer disease, frontotemporal dementia, multiple sclerosis, autism and Parkinson disease (Sandrone and Catani, 2013). In fact, DMN regions showing decreased connectivity to left and right PCC in FIDA group included regions of posterior DMN such as cuneus, PCC, and parahippocampal gyrus, is also found in a majority of patients with Alzheimer disease and other types of dementia (Chhatwal et al., 2013).

Furthermore, several studies of subjects at high risk for these disorders, including healthy relatives of patients with late onset of Alzheimer disease, have demonstrated that DMN patterns, particularly PCC and medial temporal cortex changes, are present before the disorder’s onset and could even early marker of the disease (Wang et al., 2012). For instance, a study in autosomal dominant Alzheimer disease showed alterations in DMN connectivity in both symptomatic and asymptomatic carriers of pathogenic mutations, with a higher connectivity decrease in symptomatic carriers. Of note, subtle decreases in DMN connectivity were observable before symptom onset (Chhatwal et al., 2013).

Otherwise from the field of cognitive development, individuals with attention-deficit and hyperactivity disorder—a condition related to impairment of dopamine neurotransmission system—are characterized by a decreased connectivity between precuneus and other DMN components (Somandepalli et al., 2015), and a negative relationship between the activity in dorsal anterior cingulate cortex and DMN (Castellanos et al., 2008). Correspondingly, studies in patients with schizophrenia, another illness associated with an alteration in the dopamine system, show decreased strength in DMN nodes connectivity, suggesting that these patterns could underlie symptoms such as impaired self-awareness (Dodell-Feder et al., 2014). A recent study in healthy subjects compared the neurological soft signs (NSS) score and the grade of connectivity among cortical and subcortical areas related to motor function. The results showed a negative correlation between NSS score (lower score means better performance) and resting state-activity of right posterior cingulate. NSS have been found in psychiatric disorders of neurodevelopmental origin such as schizophrenia, autism and borderline personality, among others conditions that involve dopamine and myelin alterations (Thomann et al., 2015).

Throughout brain development network properties increase integration and decrease segregation, thus WM maturation might play an important role in circuit formation or changes in their strengths (Hagmann et al., 2010). There are several studies showing a close relationship and a highly complex interaction between structural connectivity (myelin tracts) and functional connectivity (Greicius et al., 2009). Based on these results we speculate that a derailment in myelination process may reshape the connectivity map and explain in part, our finding of a connectivity absence in the posterior region of DMN in FIDA participants.

The effects of IDA in infancy on dopamine neurotransmission system could be explore assessing the executive functions, i.e., inhibitory control, set-shifting and planning, high cognitive abilities that relies on the integrity of frontostriatal circuits, in which the dopaminergic system plays a key role. At 10 years old, FIDA participants from our longitudinal study, showed a mild impairment performing an inhibitory motor task (Algarín et al., 2013). A follow-up study performed in Costa Rica indicated that FIDA at 19 years presented greater difficulty performing executive functions test (Lukowski et al., 2010). In the present study at 22 years old, a decreased DMN connectivity characterized FIDA subjects. Therefore, it is reasonable to argue that different DMN patterns in FIDA young adults might also refer to a dopaminergic dysregulation.

The MFG—another key node of DMN (Passow et al., 2015)—is involved in decision making and reasoning processes (Talati and Hirsch, 2005). In the present study the FIDA group demonstrated increased connectivity to the left MFG including DMN and dorsal attention networks, primarily included intraparietal lobule and precuneus. Sang et al. (2015) reported greater nodal centrality in MFG and parietal lobule regions in patients with Parkinson disease and proposed that these areas are highly connected to compensate for deficits caused by the disease.

Given that we did not perform a task based fMRI, it is not possible to determine whether the increased connectivity between brain circuits at rest relates to a decreased activation or suppression of DMN during tasks requiring higher cognitive resources. However, we could suggest that differences in intrinsic functional organization in the resting state, may contribute to change the interactions among specific brain regions while performing a task.

Finally, a recent study in preschool age children showed that early life stressful events are associated with greater activity of rs-fMRI in left middle frontal gyrus, independent of other variables as poverty, violence exposure and exposure to a traumatic event (Demir-Lira et al., 2016). These results provide further support for the hypothesis that environment factors like micronutrient deficits (IDA), could generate persistent effects on neurofunctional domains, including resting state connectivity, more than 20 years after the nutritional insult (Lozoff et al., 2003).

An important limitation for the interpretation of the results is the lower spatial resolution of axial slicing of rs-fMRI and the small sample size. Notwithstanding, differences in brain connectivity between groups in specific regions were significant. The correlation analysis among connectivity results and behavioral tasks are not presented in this manuscript. Given the small sample size, one of our goals is to increase the number of participants to correlate rs-fMRI findings with behavioral results in future studies.

In conclusion, the results of the rs-fMRI study show altered brain connectivity patterns in otherwise healthy FIDA young adults. Based on the hypothesis of long-lasting effects of IDA in infancy on myelination and dopaminergic system functioning, it should be possible to design studies with specific fMRI imaging measures known to depend on these systems and processes. Whether altered brain connectivity in FIDA participants influences the progression of specific cognitive functions and neurodegenerative diseases with advancing age is part of our ongoing research challenges.

## Author Contributions

CA conceptualized and designed the study, drafted the initial manuscript, and participated in critical revision of the manuscript. KDK carried out the initial analyses, and participated in critical revision of the manuscript. SR and CM led the participant recruitment, coordinated and supervised data collection and participated in critical revision of the manuscript. BL and PP conceptualized the study and critically reviewed the manuscript. BB designed the data collection tools, conceptualized the study and critically reviewed the manuscript. All authors approved the final manuscript as submitted.

## Funding

This study received a grant from the National Institutes of Health, Bethesda, MD, USA (R01 HD33487). The content of this article is solely the responsibility of the authors and does not necessarily represent the official views of the National Institute of Child Health and Human Development or the National Institutes of Health.

## Conflict of Interest Statement

The authors declare that the research was conducted in the absence of any commercial or financial relationships that could be construed as a potential conflict of interest.
